# Loss of miR-542-3p enhances IGFBP-1 expression in decidualizing human endometrial stromal cells

**DOI:** 10.1038/srep40001

**Published:** 2017-01-04

**Authors:** Hideno Tochigi, Takeshi Kajihara, Yosuke Mizuno, Yumi Mizuno, Shunsuke Tamaru, Yoshimasa Kamei, Yasushi Okazaki, Jan J Brosens, Osamu Ishihara

**Affiliations:** 1 Department of Obstetrics and Gynecology, Saitama Medical University, 38 Morohongo, Moroyama, Iruma-gun, Saitama, Japan; 2 Division of Functional Genomics and Systems Medicine, Research Center for Genomic Medicine, Saitama Medical University, 1397-1 Yamane, Hidaka city, Saitama, Japan; 3 Division of Translational Research, Research Center for Genomic Medicine, Saitama Medical University, 1397-1 Yamane, Hidaka city, Saitama, Japan; 4 Division of Reproductive Health, Warwick Medical School, Clinical Sciences Research Laboratories, University Hospital, Coventry CV2 2DX, UK; 5 Tommy’s National Miscarriage Research Centre, University Hospital Coventry and Warwickshire, Coventry CV2 2DX, UK

## Abstract

Endometrial decidualization represents an essential step for the successful implantation of the embryo; however, the molecular mechanism behind this differentiation process remains unclear. This study aimed to identify novel microRNAs (miRNAs) involved in the regulation of decidual gene expression in human endometrial stromal cells (HESCs). An *in vitro* analysis of primary undifferentiated and decidualizing HESCs was conducted. HESCs were isolated from hysterectomy specimens from normally cycling premenopausal women with uterine fibroids, who were not on hormonal treatment at the time of surgery. Primary HESCs were expanded in culture and decidualized with 8-bromo-cyclic adenosine monophosphate and medroxyprogesterone acetate. Microarray analysis identified six miRNAs differentially expressed in response to decidualization of HESCs. All but one miRNA were downregulated upon decidualization, including miR-542-3p. We demonstrated that miR-542-3p overexpression inhibits the induction of major decidual marker genes, including *IGFBP1, WNT4* and *PRL*. In addition, miR-542-3p overexpression inhibited the morphological transformation of HESCs in response to deciduogenic cues. A luciferase reporter assay confirmed that the 3′-untranslated region of *IGFBP1* mRNA is targeted by miR-542-3p. The results suggest that miR-542-3p plays an important role in endometrial decidualization by regulating the expression of major decidual marker genes.

Initiation of pregnancy represents the successful combination of two independent processes, i.e. embryo development and endometrial differentiation. Decidualization denotes the morphological and biochemical transformation of the human endometrial stromal cells (HESCs) into decidual cells, which regulate blastocyst implantation and subsequent placenta formation^1^,^2^. In contrast to many other mammals, decidualization of HESCs is independent of the presence of an implanting blastocyst and is initiated during the mid- to late-luteal phase of the cycle. The most striking morphological alteration of the decidual process is the dramatic transformation of the endometrial stromal fibroblasts into secretory epithelioid decidual cells^3^. At a molecular level, decidual transformation involves extensive reprogramming of many cell functions, mediated by the activation of key transcription factors, including progesterone receptor, forkhead box protein O1 (FOXO1), CCAAT/enhancer-binding protein beta (C/EBPβ) and the homeobox proteins HOXA10 and HOXA11. The convergence of these transcription factors on promoters of key genes drives the expression and secretion of major decidual factors, including prolactin (PRL), WNT4 and insulin-like growth factor binding protein-1 (IGFBP-1)^3^,^4^.

MicroRNAs (miRNAs) are single-stranded small non-coding RNAs of approximately 22 nucleotides that regulate gene expression by blocking the translation or by decreasing the stability of mRNAs^5^. In mammals, miRNAs are predicted to control the activity of approximately 50% of all protein-coding genes^6^,^7^. The majority of mammalian microRNAs are spatiotemporally regulated. Accumulating evidence has implicated miRNAs in the regulation of numerous biological processes, ranging from proliferation, differentiation and apoptosis to embryo development and implantation^8^. In addition, aberrant miRNA expression is a feature of many pathological conditions, including cancer, viral infections, metabolic diseases and neurological disorders^9^. However, our understanding of the expression, regulation and function of miRNAs in differentiating HESCS remains limited. Hence, we employed both miRNA and gene expression microarrays to identify miRNAs and target genes that are regulated upon decidual transformation of HESCs. We show that miR-542-3p is a potent negative regulator of HESC differentiation in response to deciduogenic cues.

## Results

### Differentially expressed miRNAs upon decidualization

The efficiency of decidualization in primary HESC cultures was confirmed by observing morphological changes and by measuring PRL secretion, the principal decidual marker^10^,^11^. HESCs exhibited a spindle-shaped fibroblast-like appearance when propagated in culture but acquired the typical morphology of decidual cells, i.e. an enlarged cell size with larger nuclei and abundant cytoplasm, upon treatment with 8-bromo-cyclic adenosine monophosphate (8-br-cAMP) and medroxyprogesterone acetate (MPA) for 6 days (Fig. S1A). Furthermore, PRL secretion markedly increased in response to the treatment with 8-br-cAMP and MPA for 6 days (Fig. S1B).

**Figure 1:**
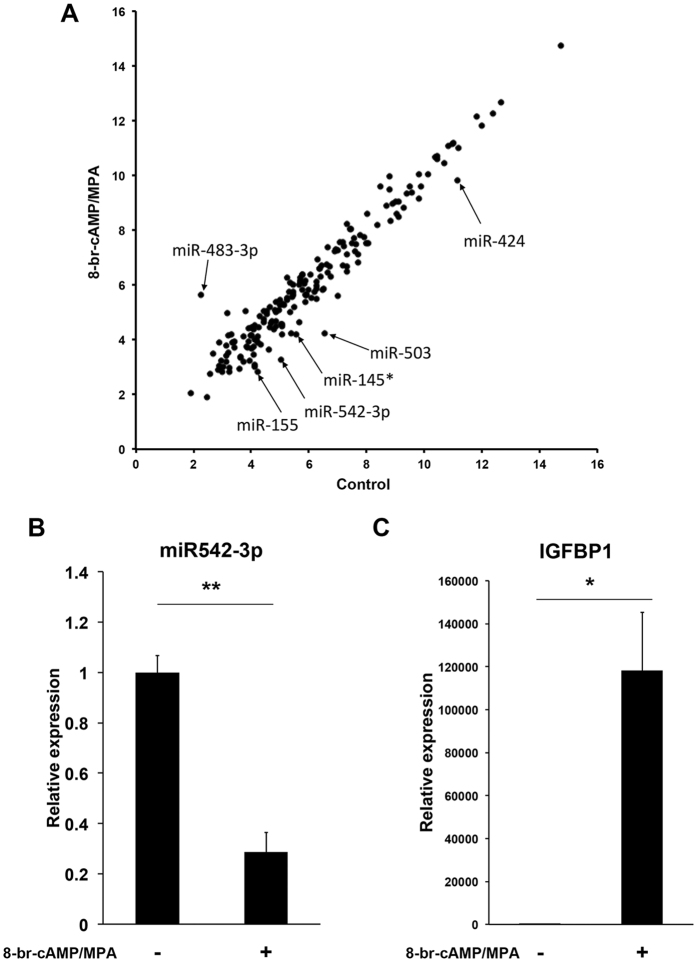
Identification of miRNAs involved in decidualization. (**A**) Scatter plot of miRNA microarray results. Total RNA from three independent HESC cultures, decidualized or not, were labelled and hybridised separately on the Agilent miRNA microarray, and signal intensity, which corresponds to the expression level of each miRNA, was measured. The intensity was averaged and converted to a log2 value. Expression level of each miRNA is shown as a scatter plot. The level of expression in undifferentiated and decidualizing HESCs is shown on the Y-axis and X-axis, respectively. qRT-PCR analysis of miR-542-3p and *IGFBP1* mRNA expression in undifferentiated and differentiating HESCs in samples used for microarray experiments. (**B**) The level of miR-542-3p (miR-542-3p/*U6* ratio) expression in HESCs treated with 8-br-cAMP and MPA for 6 days was significantly down-regulated in comparison to untreated HESCs. (**C**) *IGFBP1* mRNA expression, normalized to *GAPDH* mRNA (glyceraldehyde phosphate dehydrogenase), in HESCs treated with 8-br-cAMP and MPA for 6 days was significantly up-regulated in comparison to undifferentiated HESCs. The experiments were performed using three independent primary cell cultures. The data are shown as mean ± SEM. **P < 0.01.

To identify miRNAs differentially expressed after 6 days of differentiation, 3 paired undifferentiated and decidualizing HESC cultures were subjected to miRNA microarray analysis. miRNAs consistently upregulated (>2-fold) or downregulated (<0.5-fold) in pair-wise comparison of the 3 biological repeat experiments were extracted. Among the 1,205 sets of human miRNAs covered by the array, five (miR-503, miR-542-3p, miR-155, miR-145* and miR-424) were significantly down-regulated upon decidualization (<0.5-fold) (Table 1A), whereas a single miRNA (miR-483-3p) increased in expression (>2-fold) (Table 1B) in all three decidualizing primary HESC cultures (Fig. 1A and Table 1A and B).

### Identification of decidual target genes

Hundreds of candidate target genes for each differentially expressed miRNA were predicted on public miRNA databases. The expression of most of these putative target genes will be down-regulated upon binding of miRNA to complementary seed sequences in target transcripts^6^,^7^. To identify miRNA target genes relevant to decidualization, we performed gene expression microarrays on the same RNA samples used in miRNA array analysis. This analysis identified six major induced decidual genes that were also putative targets of miR-542-3p, miR-424 or miR-503 (Table 2). We reasoned that induction of these decidual genes could be dependent upon the down-regulation of targeting miRNAs. We then focused on miR-542-3p and its putative target *IGFBP1*, which encodes for the major decidual secretory protein^4^,^12^. Differential expression of miR-542-3p was confirmed by quantitative real-time polymerase chain reaction (qRT-PCR) analysis (Fig. 1B). qRT-PCR analysis also confirmed the marked induction of *IGFBP1* in HESCs treated with 8-br-cAMP and MPA (Fig. 1C).

### Decidualisation is inhibited by miR-542-3p

**Figure 2:**
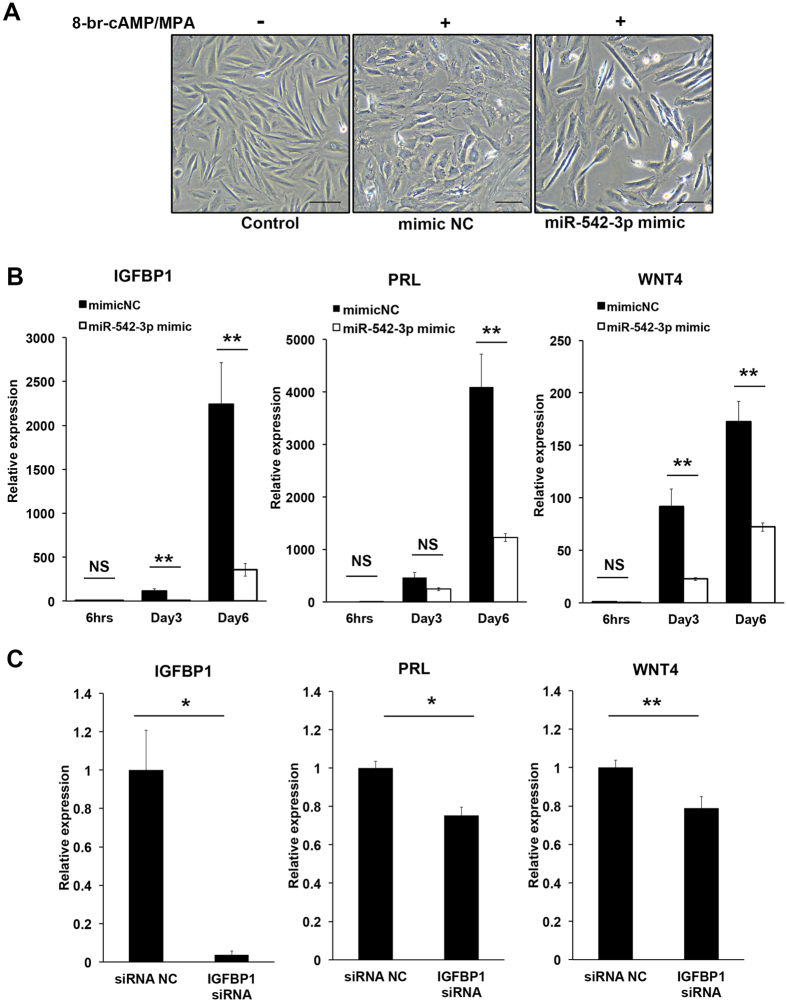
Effect of miR-542-3p overexpression or IGFBP-1 knockdown in decidualizing HESCs. (**A**) Morphological changes of HESCs transfected with miR-542-3p mimic. HESCs transfected with negative control miRNA mimic (NC mimic) showed morphological changes characteristic of decidual cells upon treatment with 8-br-cAMP and MPA for 6 day. By contrast, morphological transformation was absent upon transfection of cells with miR-542-3p mimic. Scale bars = 100 μm. (**B**) qRT-PCR analysis of *IGFBP1, PRL* and *WNT4* transcript levels in HESCs transfected with a miR-542-3p mimic or NC mimic and decidualized for. 6 days. Expression levels were normalised to *GAPDH*. Data represent mean ± SEM of three independent experiments. (**C**) qRT-PCR analysis *IGFBP1, PRL* and *WNT4* transcript levels in HESCs transfected with siRNA targeting IGFBP-1 and then decidualized for 6 days. Expression levels were normalised to *GAPDH*. Data are the mean ± SEM of three independent experiments **P < 0.01, *P < 0.05.

To determine the functional significance of the loss of miR-542-3p expression in differentiating HESCs, primary cultures were transfected with either miR-542-3p mimic or negative control miRNA mimic prior to decidualization over a time course lasting 6 days. To assess potential toxicity of transfection, cell apoptosis/necrosis detection assay was performed. The abundance of apoptotic/necrotic cells did not differ between the experimental groups (*P* > 0.05; Fig. S2). In cells transfected with negative control miRNA mimic, differentiating HESCs displayed distinct morphological characteristics, whereas this response was completely lacking in cultures transfected with the miR-542-3p mimic (Fig. 2A). To evaluate transfection efficiency, we have performed qRT-PCR for miR542-3p. Compared with cells transfected with control miRNA mimic, the expression of miR-542-3p in response to 8-br-cAMP and MPA treatment was markedly up-regulated, approximately 2,200-fold, in cultures expressing miR-542-3p mimic (Fig. S3). The expression of *IGFBP1* was also evaluated using qRT-PCR analysis. The expression of *IGFBP1* in HESCs treated with 8-br-cAMP and MPA increased in a time-dependent manner (Fig. 2B). Compared to cells transfected with control miRNA mimic, the induction of *IGFBP1* transcripts in response to 8-br-cAMP and MPA treatment was markedly inhibited in cultures transfected with the miR-542-3p mimic (Fig. 2B). The expression of the other decidual markers was also examined. Similar to *IGFBP1*, the induction of *PRL* and *WNT4* was repressed by miR-542-3p mimic (Fig. 2B). To clarify whether inhibition of *PRL* and *WNT4* expression was a direct effect of miR-542-3p or an indirect effect caused by *IGFBP1* knockdown, we transfected primary cultures with either non-targeting small interfering RNA (siRNA) or siRNA targeting *IGFBP1*. Following transfection, the HESC cultures were decidualized for 6 days. Similar to miR-542-3p mimic transfection, *IGFBP1* knockdown was sufficient to impair *PRL* and *WNT4* expressions (Fig. 2C), suggesting that IGFBP-1 itself plays a role in regulating other decidual marker genes. Further, the decrease in IGFBP-1 secretion by miR-542-3p was confirmed by an enzyme-linked immunosorbent assay (ELISA) assay of the culture supernatants (Fig. S4). Taken together, these results demonstrate that miR-542-3p is a potent inhibitor of HESC differentiation.

### *IGFBP1* is a *bona fide* target of miR-542-3p

**Figure 3:**
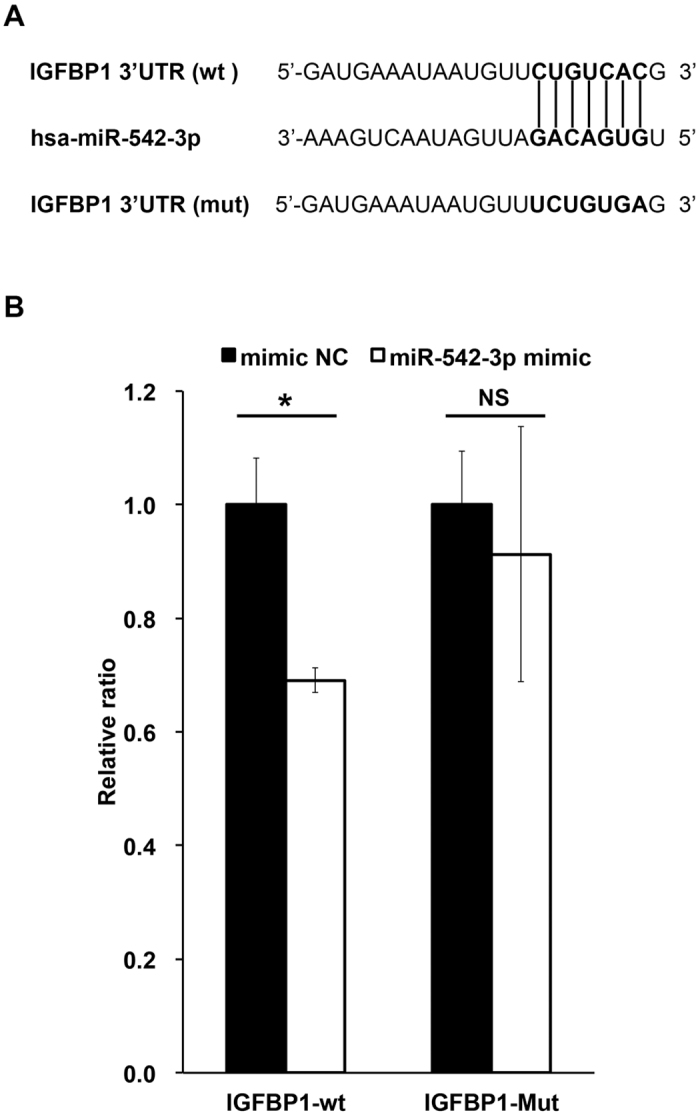
*IGFBP1 is* a direct target of miR-542-3p. (**A**) The predicted target sequence of miR-542-3p in the 3′ untranslated region (UTR) of IGFBP1 and the mutated sequence used in the luciferase assay are shown. (**B**) Relative luciferase activity of IGFBP1-wt and IGFBP1-mut reporter vectors co-transfected with miR-542-3p mimic (miR-542-3p) or negative control (NC) miRNA in decidualized HESCs. Using IGFBP1-wt vector, the relative luciferase activity was decreased by 31% upon transfection with miR-542-3p compared to NC mimic. The IGFBP1-mut reporter was not significantly inhibited by miR-542-3p mimic. Data are shown as the mean ± SEM of three independent experiments. *P < 0.05; NS, Not significantly different.

To examine if miR-542-3p targets *IGFBP1* directly, a reporter construct was generated by inserting the putative binding site of miR-542-3p into the 3′ untranslated region (UTR) of the *IGFBP1* gene downstream of a firefly luciferase reporter vector (IGFBP1-wt). The seed sequence of the target site was also mutated to generate a control plasmid (IGFBP1-mut) (Fig. 3A). As shown in Fig. 3B, luciferase activity was decreased upon co-transfection of decidualized HESCs with the IGFBP1-wt reporter construct and miR-542-3p mimic when compared to co-transfection with a control miRNA mimic (*P* < 0.05). In contrast, no differences were found when the cells were transfected with the IGFBP1-mut reporter. Taken together, the data confirms that miR-542-3p inhibits *IGFBP1* expression by binding to its 3′ UTR.

## Discussion

In the current study, we combined miRNA and mRNA microarray analyses of primary HESC cultures to identify novel decidual regulators. We identified five miRNAs robustly repressed and a single miRNA consistently induced upon decidual transformation of HESCs. Parallel transcriptional profiling enabled us to identify the miRNA–mRNA pairs that functionally regulate decidualization. We focused on miR-542-3p and its predicted target gene *IGFBP1*, a well-known decidual marker^4^,^12^, for further analysis. We were able to demonstrate that miR-542-3p directly regulates *IGFBP1* expression during HESCs and indirectly regulates the expression of other decidual marker genes, such as *PRL* and *WNT4*.

As small non-coding RNAs, miRNAs are classified as regulatory RNAs and have been reported to have various roles, including cell growth, proliferation and differentiation^8^. Because each miRNA is predicted to target a broad range of mRNAs based on the degree of sequence homology, the expression of approximately 50% of all protein-coding genes are estimated to be subjected to miRNA regulatory function^6^,^7^. The inverse relationship between the expression of miRNAs and their target genes is complex and evolving. Notably, aberrant miRNA expression has been associated with endometrial disorders, including endometriosis, endometrial hyperplasia and carcinoma^13^,^14^. On the other hand, it has been reported that miR-542-3p is generally underexpressed in cancers arising from the colon, prostate and lung. Further, overexpression of miR-542-3p can inhibit cell growth and prevent tumour formation *in vivo*^15^. These observations suggest that miR-542-3p is a promising target for cancer therapy. On the other hand, Kureel *et al*. demonstrated that miR-542-3p suppresses osteogenic differentiation and promotes osteoblast apoptosis by repressing bone morphogenetic protein 7 and its downstream signalling^16^. To the best of our knowledge, the current report is the first to identify that miR-542-3p directly regulates morphological and biological differentiation of HESCs through targeting *IGFBP1* expression.

We and others previously reported that Dicer knockdown does not block HESCs decidualization^17^,^18^. Female mice lacking Dicer in the reproductive tract are infertile due to defective oviducts but exhibited histologically normal decidual responses. Therefore, we speculated that miRNAs may primarily serve to prevent premature or illicit activation of decidual genes. Endometrial expression of miRNAs upon decidualization has been studied in few other species and, as aforementioned, some functional studies have been carried out in rodents^19^,^20^,^21^. Members of three miRNAs families, including Let-7 Family, miR-200 Family, and miR-30 Family, are frequently identified as differentially expressed in these studies^22^. To the best of our knowledge, the current report is the first to identify that miR-542-3p directly regulates morphological and biological differentiation of HESCs through targeting *IGFBP1* expression.

Uterine expression of cyclooxygenase-2 is regulated by miR-101a and miR-199a* at implantation in mice^23^. Expression profiling of miRNAs during the menstrual cycle demonstrated that they are regulated by ovarian hormones^18^,^24^. Further, recent reports demonstrate that aberrant miRNA expression is associated with reproductive disorders, including endometriosis, endometrial hyperplasia and carcinoma^13^,^14^. The present study identified six miRNAs differentially expressed in response to decidualization of HESCs. On the other hand, Estella *et al*.^17^ found a total of 26 and 17 miRNAs up- and down-regulated, respectively, upon decidualization. Among these miRs, only miR-155 was identified as an induced miRNA upon decidualization in both studies. Further, Estella *et al*. found no significant change in the expression of miR-542-3p, which may be due to the differences in endometrial samples, decidualization protocol, or the expression profiling technique. Perhaps most notable, decidualization in the study of Estella *et al*. was induced by a combination of estradiol and progesterone, which is a much weaker HESCs differentiation stimulus than treatment with a cAMP analogue and MPA^25^,^26^.

IGFBP-1 is widely used as phenotypic markers of decidualization but its involvement in the regulation of this transformational process has been largely ignored. Decidual IGFBP-1 is thought to have IGF-dependent and IGF-independent actions on trophoblast invasion at implantation^12^. Importantly, exposure of HESCs to recombinant human IGFBP-1 has been reported to promote morphological decidual transformation and increase PRL secretion by interacting with α5β1 integrin expressed on the surface of HESCs^27^. These observations strongly infer an auto/paracrine role IGFBP1 in enhancing decidual transformation of HESCs. Transfection of HESCs with miR-542-3p inhibited *PRL* and *WNT4* expression after 6 days of decidualization by 60–70%. However, transfection of cells with siRNA targeting *IGFBP1* decreased *PRL* and *WNT4* mRNA levels much more modestly (∼20%). This discrepancy may reflect the ability of a single miR to target multiple mRNAs and, conversely, that a single mRNA can be targeted by multiple miRs^28^. Therefore, repression of *PRL* and *WNT4* expression in decidualizing HESCs upon transfection with miR-542-3p mimic likely involves mechanisms other than the inhibition the auto/paracrine actions of IGFBP-1. Clearly, additional experiments are required to test this hypothesis.

Our study has several limitations. Although the decidualization of primary HESCs is a well-established model, the absence of other cellular constituents of the endometrium, e.g. epithelial or vascular cells, may impact on the results. Furthermore, all primary cultures were established from patients requiring surgery for uterine fibroids as it is challenging to obtain biopsies from women who are free of gynaecological diseases. Although the current study focused on miR-542-3p, other differentially expressed miRNAs, including miR-424, miR-503 and miR-155, may equally functionally regulate decidualization. Interestingly, it has been reported that miR-424 and miR-503 are derived from a polycistronic precursor miR-424-503. These miRNAs were reported to have combinatorial effects within the cellular system^29^. It is possible that miR-424 in cooperation with miR-503 coordinates endometrial differentiation. Although only miR-542-3p was intensively investigated in the present study, the detailed roles of other miRNAs should be explored in a future study.

In conclusion, the present study demonstrates that the downregulation of miR-542-3p expression in HESCs is permissive for morphological and molecular differentiation of HESCs. Our results indicate that miR-542-3p plays an important role in endometrial decidualization, at least in part, by regulating *IGFBP1* expression.

## Methods

### Experimental ethics policy

The protocol of the current study was approved by the Institutional Review Board of the Saitama Medical University Hospital (11-017-1). This study was carried out in accordance with the approved guideline. Written informed consent was obtained from all participating subjects.

### Tissue collection and isolation of HESCs

HESCs were obtained at the time of hysterectomy for uterine fibroids from normally cycling premenopausal women. Patients were not undergoing hormonal treatment at the time of surgery. All the samples were collected during the proliferative phase of the cycle. HESCs were isolated and cultured as previously described^30^,^31^,^32^. Briefly, endometrial samples were collected in Dulbecco’s modified Eagle’s medium (DMEM)/F-12 containing Antibiotic-Antimycotic solution (Invitrogen). After enzymatic digestion by DNase I (Sigma) and Collagenase I a (Sigma), the stromal cells were separated from epithelial cells and passed into culture. Proliferating HESCs were cultured in maintenance medium of DMEM/F-12 containing 10% dextran-coated charcoal-treated FBS and 1% antibiotic–antimycotic solution (Life technologies). Confluent monolayers of HESCs were treated with or without 0.5 mM 8-bromo-cyclic adenosine monophosphate (8-br-cAMP; Sigma) and 10^−6^ M medroxyprogesterone acetate (MPA; Sigma). All experiments were conducted before the third passage of the cultures.

### Measurement of PRL and IGFBP1

The concentration of PRL in the HESC culture media was measured by microparticle enzyme immunoassay (AxSYM system; Abbott Laboratories, North Chicago, IL). The level of IGFBP1 in culture media was determined using a sandwich-type immunoassay (Mediagnost, Reutlingen, Germany). The assay was performed in triplicate or duplicate and normalised to the total protein content of cultures.

### RNA extraction

Total RNA was purified from HESCs using the miRNeasy Mini kit (Qiagen, Valencia, CA, USA) according to the manufacturer’s protocol. RNA quality was verified by measuring the absorbance at 230 nm, 260 nm and 280 nm using a NanoDrop spectrometer (NanoDropTechnology, San Diego, CA, USA).

### miRNA expression array

The analysis of the miRNA microarray was performed using a commercial human miRNA microarray (G4872A Human Rel. 16.0, Agilent Technologies, Santa Clara, CA, USA), which included probes for 1,205 human and 144 viral miRNAs based on Sanger miRBase Release 16.0. Total RNA samples from three independent, undifferentiated and decidualizing HESC cultures, were subjected to miRNA microarray. Using 100 ng of total RNA of each of the 6 samples, miRNAs were labelled and hybridised onto the miRNA microarray using the miRNA Complete Labelling and Hybridisation Kit (Agilent Technologies), according to the manufacturer’s protocol. After hybridisation for 22 h, the miRNA array was washed and the signal intensity of an individual miRNA probe spot was measured using a microarray scanner (G2505B; Agilent Technologies) and quantified with Feature Extraction ver. 10.7.3.1 software (Agilent Technologies) (Supplementary excel file 1). The 6 data sets were normalised using a quantile normalisation method^33^ (Supplementary Table S1). Expression of miRNAs was analysed pair-wise in undifferentiated and decidualising cells in the 3 biological repeat experiments. Upregulated and downregulated miRNAs were defined, respectively, by >2-fold or <0.5-fold change in expression level upon decidualization in pair-wise analysis of each of the 3 repeat experiments. The fold-change values of miRNAs were then calculated as average fold-change value from three independent paired cultures. *P* value was calculated by two-tailed Student’s *t*-tests.

### Gene expression profiling

The RNA samples subjected to miRNA microarray analysis were also used to identify putative miRNA target genes. Briefly, total RNA from the 3 undifferentiated and 3 decidualized cultures were combined separately; and the two sets of mRNAs subjected to gene expression microarrays. Total RNA was reverse-transcribed, amplified and labelled using a GeneChip^®^ 3′IVT Expression Kit (Affymetrix Inc., Santa Clara, CA, USA). The complementary RNA (cRNA) was purified, fragmented according to the manufacturer’s protocol and hybridised on the Affymetrix GeneChip^®^ Human Genome U133 Plus 2.0 array. Hybridisation, washing, staining and scanning were performed using the Affymetrix GeneChip^®^ system instruments and protocols. The expression levels of each gene obtained from the scanned data were normalised among samples using the robust multi-array average (RMA) method. The mRNAs that were differentially upregulated (>2-fold) or downregulated (<0.5-fold) upon decidualization were extracted.

### qRT-PCR for miRNAs

To confirm the relative expression level of objective miRNA, total RNA was subjected to qRT-PCR analysis. The TaqMan microRNA RT kit (Applied Biosystems, Carlsbad, CA, USA) was used to synthesise cDNA from 5 ng of RNA. The PikoReal 96 Real-Time PCR system (Thermo Fisher Scientific Inc., Waltham, MA, USA) and TaqMan Universal Master Mix II with UNG (Applied Biosystems) were used to perform qRT-PCR in duplicate. TaqMan MicroRNA assays (Applied Biosystems) for miR-542-3p (Assay ID: MC001284) and U6 (as endogenous control) were used as primers for reverse transcription and amplification. The mature sequences of hsa-miR-542-3p were 5′-UGUGACAGAUUG AUAACUGAAA-3′ (miRBase Accession number: MIMAT0003389). The expression level of miR-542-3p relative to U6 was calculated by the 2^−ΔΔct^ method^34^.

### qRT-PCR for mRNAs

Total RNAs of each sample were reverse transcribed using BioScript reverse transcriptase (Bioline, London, UK) with oligo dT primers, and cDNAs obtained were mixed with gene specific primers and Power SYBR Green PCR Master Mix (Applied Biosystems) and subjected to the qPCR analysis using the MX3000p system (Stratagene, La Jolla, CA, USA) or PikoReal 96 Real-Time PCR system. Primer sequences for each genes were: 5′-CGACCACTTTGTCAAGCTCA-3′ [glyceraldehyde phosphate dehydrogenase (*GAPDH)*-forward], 5′-AGGGGTCTACATGGCAACTG-3′ (*GAPDH*-reverse); 5′-CTGCGTGCAGGAGTCTGA-3′ (*IGFBP1*-forward), 5′-CCCAAAGGATGGAATGATCC-3′ (*IGFBP1*-reverse); 5′-CTACATCCATAACCTC TCCTCA-3′ (*PRL*-forward), 5′-GGGCTTGCTCCTTGTCTTC-3′ (*PRL*-reverse); 5′*-CAT GCAACAAGACGTCCAAG*-3′ (*WNT4-*forward), 5′-AAGCAGCACCAGTGGAATTT-3′ (*WNT4*-reverse). *IGFBP1, PRL* and *WNT4* expression levels, relative to *GAPDH,* were calculated using the 2^−ΔΔct^ method.

### Identification of differentially expressed miRNAs

Array data for miRNA and mRNA were uploaded to the Ingenuity Pathways Analysis software (Ingenuity^®^ Systems; www.ingenuity.com). Predicted target genes of differentially expressed miRNAs were identified using TargetScan version 6.2 and the Ingenuity^®^ Knowledge Base and filtered on the basis of the direction of regulation of putative target genes in undifferentiated and decidualizing HESCs. The data for the differentially expressed mRNAs were integrated with miRNA expression data, using the IPA expression pairing function to obtain miRNA-mRNA relationships that showed expression change in the opposite directions.

### Transfection of miRNA mimics and siRNA

Immediately prior to transfection, the culture medium of HESCs was changed to antibiotic-free decidualization medium containing 0.5 mM 8-br-cAMP and 10^−6^ M MPA. Control cultures were maintained at the standard medium. mirVana^TM^ miRNA mimic (#MC11340; Applied Biosystems) for hsa-miR-542-3p or siRNA targeting IGFBP1 (Silencer select, s7225; Applied Biosystems) was mixed with Lipofectamine 2000 (Invitrogen), according to the manufacturer’s instructions, and added to HESCs in six-well culture plates at a density of 80–90% confluence. Control cultures were transfected with control miRNA mimic or non-targeting siRNA. Final concentration of miRNA mimic and siRNA was 30 nM. After 6 h and 3 days, cells were subjected to a change of antibiotics media to decidual differentiation with 0.5 mM 8-br-cAMP and 10^−6^ M MPA or control media. Morphological evaluation, RNA extraction and qRT-PCR analysis were performed on day 6 of decidualization.

### Reporter assay for the evaluation of the miRNA target

The miR-542-3p candidate targeting sequence of *IGFBP1* gene (5′-GAUGAAAUAAUGUU**CUGUCAC**G-3′, part of the NCBI RefSeq ID of NM_000596) and its mutated sequence (5′-GAUGAAAUAAUGUU**UCUGUGA**G-3′) were inserted downstream of the pGL4.13 luciferase expression vector (Promega Corporation, Madison, WI, USA). The resulting constructs were named as IGFBP1-wt and IGFBP1-mut, respectively. Those two vectors with an amount of 200 ng and a final concentration of 30 nM miR-542-3p mimic were co-transfected to HESCS treated with 0.5 mM 8-br-cAMP and 10^−6^ M MPA for 6 days before transfection. As a negative control, native pGL4.13 luciferase vector was also transfected in parallel. For normalisation, 30 ng of Renilla luciferase vector, pGL4.74 (Promega), was also transfected into all samples. Transfections with all vectors and RNA combinations were repeated three times. After 48 h, the cells were harvested with PLB reagent (Promega), following which the firefly and Renilla luciferase activity was measured in each well using the dual luciferase assay kit (Promega) with the ARVO MX plate reader. The relative firefly luciferase activity was calculated by normalisation to the Renilla luciferase activity.

### Cell apoptotic/Necrotic detection assay

To assess any potential toxicity of the miR-542-3p mimic transfection, apoptosis and necrosis were quantified by direct determination of nucleosomal DNA fragmentation using the Apoptotic/Necrotic/Healthy Cells Detection Kit (Takara bio Inc., Japan) as per the manufacture’s instructions.

### Statistical analyses

All experiments were repeated a minimum of three times using independent primary cell cultures. All data were checked for their normal distribution using the Ronald-Fisher’s test, and if it was considered significant by the accuracy of *P* < 0.05, non-parametric statistical analysis was applied. Data are expressed as mean ± standard error (SE). Statistical analyses were performed by two-tailed Student’s *t*-tests. Results are presented as mean ± SE. *P* < 0.05 was considered significant.

## Additional Information

**How to cite this article**: Tochigi, H. *et al*. Loss of miR-542-3p enhances IGFBP-1 expression in decidualizing human endometrial stromal cells. *Sci. Rep.* **7**, 40001; doi: 10.1038/srep40001 (2017).

**Publisher's note:** Springer Nature remains neutral with regard to jurisdictional claims in published maps and institutional affiliations.

## Supplementary Material

Supplementary Information

Supplementary Dataset 1

## Figures and Tables

**Table 1:** Differentially expressed miRNAs upon decidualization of primary HESCs.

miRNA	Fold-change	p value
hsa-miR-503	0.20	0.0227
hsa-miR-542-3p	0.30	0.0085
hsa-miR-155	0.38	0.0074
hsa-miR-145*	0.39	0.0047
hsa-miR-424	0.40	0.0030
**(B) Up-regulated miRNA**		
hsa-miR-483-3p	10.39	0.0448

**Table 2:** Predicted decidual target genes.

miRNA	Target gene	Gene name	Fold change*^1^
miR-542-3p	*IGFBP1*	insulin-like growth factor binding protein 1	232.16
	*WNT4*	wingless-type MMTV integration site family, member 4	73.93
miR-424	*FOXO1*	forkhead box O1	10.07
	*IGF1*	insulin-like growth factor 1 (somatomedin C)	7.43
	*GADD45G*	growth arrest and DNA-damage-inducible, gamma	40.20
miR-503	*IGF2*	insulin-like growth factor 2 (somatomedin A)	8.47
	*IGF1*	insulin-like growth factor 1 (somatomedin C)	7.43

The expression of all putative target genes changed >2-fold in either direction upon decidualization.
