# Erratum: Loss of miR-542-3p enhances IGFBP-1 expression in decidualizing human endometrial stromal cells

**DOI:** 10.1038/srep46591

**Published:** 2017-04-26

**Authors:** Hideno Tochigi, Takeshi Kajihara, Yosuke Mizuno, Yumi Mizuno, Shunsuke Tamaru, Yoshimasa Kamei, Yasushi Okazaki, Jan J. Brosens, Osamu Ishihara

Scientific Reports 7: Article number: 40001; 10.1038/srep40001 published online: 01 04 2017; updated: 04 26 2017.

In this Article, the rows in Table 2 are misaligned. The correct Table 2 appears below.

## Figures and Tables

**Table 2:** 

miRNA	Target gene	Gene name	Fold change*^1^
miR-542-3p	*IGFBP1*	insulin-like growth factor binding protein 1	232.16
	*WNT4*	wingless-type MMTV integration site family, member 4	73.93
miR-424	*FOXO1*	forkhead box O1	10.07
	*IGF1*	insulin-like growth factor 1 (somatomedin C)	7.43
miR-503	*GADD45G*	growth arrest and DNA-damage-inducible, gamma	40.20
	*IGF2*	insulin-like growth factor 2 (somatomedin A)	8.47
	*IGF1*	insulin-like growth factor 1 (somatomedin C)	7.43

