# Supplementary material for: Loss of miR-542-3p enhances IGFBP-1 expression in decidualizing human endometrial stromal cells
Source: Sci Rep. 2017 Jan 4;7:40001. doi: 10.1038/srep40001 (PMC5209665; doi:10.1038/srep40001)

Supplementary Information

Loss of miR-542-3p enhances IGFBP-1 expression in decidualising human endometrial stromal cells

Hideno Tochigi[a,b], Takeshi Kajihara[a*], Yosuke Mizuno[b], Yumi Mizuno[a],

Shunsuke Tamaru[a,b], Yoshimasa Kamei[a], Yasushi Okazaki[b,c], Jan J Brosens[d,e],

Osamu Ishihara[a]

[a]Department of Obstetrics and Gynecology, Saitama Medical University, 38 Morohongo, Moroyama, Iruma-gun, Saitama, Japan

[b]Division of Functional Genomics and Systems Medicine, Research Center for Genomic Medicine, Saitama Medical University, 1397-1 Yamane, Hidaka city, Saitama, Japan

[c]Division of Translational Research, Research Center for Genomic Medicine, Saitama Medical University, 1397-1 Yamane, Hidaka city, Saitama, Japan

[d]Division of Reproductive Health, Warwick Medical School, Clinical Sciences Research Laboratories, University Hospital, Coventry CV2 2DX, UK

[e]Tommy's National Miscarriage Research Centre, University Hospital Coventry and Warwickshire, Coventry CV2 2DX, UK

Figure legends

Figure S1. Morphological transformation and PRL production in decidualizing HESCs (*A*) Undifferentiated primary HESCs exhibit a fibroblastic spindle-shaped morphology (control). Primary HESCs treated with 8-br-cAMP (0.5 mM) and MPA (10^{-6} M) for 6 days transform the spindle-shaped cells into cells with larger nuclei and abundant cytoplasm, which are the typical morphology of decidual cells (8-br-cAMP/MPA). Scale bar indicates 100μm. (*B*) Confluent HESCs were treated with or without 8-br-cAMP and MPA for 6 days. The medium was changed at day 3. The data represent the mean of PRL protein concentration in the supernatant, normalised by the total protein content. Data are shown are mean ± SEM of five individuals. $*P < 0.05$

Figure S2. Cell death analysis in response to transfection in with miR-542-3p mimic or negative control (NC) mimic. (*A*) Green color indicates apoptotic cells, magenta color indicates necrotic cells, and blue color indicates nuclear. Scale bar indicates 100μm. (*B*) Four fields of fluorescence microscopy images of each condition were analysed. Data are shown as mean ± SEM of three individuals NS indicates no significant.

Figure S3. To evaluate the transfection efficiency, we have performed qRT-PCR for

miR542-3p. Compared with cells transfected with control miRNA mimic, the expression of miR-542-3p in response to 8-br-cAMP and MPA treatment was markedly up-regulated, approximately 2,200-fold, in cultures first transfected with miR-542-3p mimic. Data depict mean ± SEM of three individuals ***$P < 0.001$

Figure S4. IGFBP-1 secretion by decidualized HESCs first transfected with a miR-542-3p mimic was measured using ELISA. Data represent mean ± SEM of three individuals **$P < 0.01$

Supplementary Table S1

	Expression intensity (non-treated)				Expression intensity (treated)				Average ratio	
	4(-)	5(-)	6(-)	Average	4(+)	5(+)	6(+)	Average	Treated/non-treated	ttest
hsa-miR-503	78.49	123.05	81.67	94.40	8.34	27.38	21.00	18.91	0.20	0.0227
hsa-miR-542-3p	39.84	29.26	28.95	32.68	4.68	12.06	12.40	9.71	0.30	0.0085
hsa-miR-155	20.10	15.67	20.31	18.69	8.18	7.30	5.76	7.08	0.38	0.0074
hsa-miR-335	190.64	109.46	82.84	127.65	50.24	52.13	44.02	48.80	0.38	0.1347
hsa-miR-145*	54.65	47.28	41.60	47.84	16.30	23.41	15.67	18.46	0.39	0.0047
hsa-miR-424	2,431.51	2,330.09	2,117.75	2,293.11	1,214.25	762.30	762.30	912.95	0.40	0.0030
hsa-miR-542-5p	42.54	42.54	39.84	41.64	14.87	21.79	20.10	18.92	0.45	0.0032
hsa-miR-1181	25.22	12.40	14.21	17.28	6.33	7.71	10.01	8.02	0.46	0.1393
hsa-miR-424*	18.07	21.00	13.87	17.65	9.21	9.21	6.86	8.43	0.48	0.0340
hsa-miR-450a	56.92	52.13	43.17	50.74	22.14	28.20	24.82	25.05	0.49	0.0129
hsa-miR-495	21.00	24.58	27.75	24.44	8.48	15.98	13.21	12.56	0.51	0.0159
hsa-miR-143	224.22	190.64	216.04	210.30	92.32	129.43	119.76	113.84	0.54	0.0031
hsa-miR-574-5p	29.26	33.01	38.72	33.66	18.88	19.62	16.76	18.42	0.55	0.0230
hsa-miR-1274b	141.23	209.99	131.40	160.88	82.84	105.11	78.49	88.81	0.55	0.0881
hsa-miR-10a	129.43	49.82	52.13	77.13	57.87	36.52	41.12	45.17	0.59	0.3460
hsa-miR-1274a	16.76	18.07	11.13	15.32	9.80	10.01	8.64	9.48	0.62	0.1058
hsa-miR-145	1,039.44	836.46	836.46	904.12	447.53	735.53	504.80	562.62	0.62	0.0404
hsa-miR-3665	112.99	92.32	64.46	89.92	67.35	59.60	41.60	56.18	0.62	0.1231
hsa-miR-505	15.67	11.13	9.47	12.09	8.64	7.97	6.33	7.64	0.63	0.1256
hsa-miR-376a	114.44	80.05	80.05	91.51	35.09	63.20	75.51	57.93	0.63	0.1127
hsa-miR-376c	176.01	151.19	151.19	159.47	69.02	117.71	117.71	101.48	0.64	0.0506
hsa-miR-16	656.47	541.38	485.40	561.08	336.82	426.17	322.66	361.88	0.64	0.0370
hsa-miR-3195	19.28	16.57	14.87	16.91	12.40	11.85	8.79	11.01	0.65	0.0266
hsa-miR-4281	279.98	158.37	195.87	211.41	190.64	109.46	114.44	138.18	0.65	0.1820
hsa-miR-31	32.16	33.60	35.81	33.86	15.35	29.26	22.14	22.25	0.66	0.0932
hsa-miR-181b	44.02	82.84	78.49	68.45	29.78	60.50	46.68	45.66	0.67	0.2138
hsa-miR-762	57.87	41.12	29.26	42.75	33.60	22.55	29.78	28.64	0.67	0.2253
hsa-miR-125a-3p	7.30	6.33	2.83	5.49	2.83	2.83	5.44	3.70	0.67	0.3395
hsa-miR-134	15.19	13.42	11.85	13.49	7.71	10.47	9.21	9.13	0.68	0.0269
hsa-miR-625	10.47	7.44	8.79	8.90	6.86	5.76	5.92	6.18	0.69	0.0750

hsa-miR-181a	181.76	322.66	292.49	265.64	114.44	254.45	185.55	184.81	0.70	0.2421
hsa-miR-20b	20.31	19.28	20.65	20.08	11.85	14.87	15.35	14.02	0.70	0.0206
hsa-miR-181a-2*	17.82	37.61	44.90	33.44	19.28	21.50	30.71	23.83	0.71	0.3626
hsa-miR-15b	426.17	426.17	504.80	452.38	426.17	292.49	254.45	324.37	0.72	0.1171
hsa-miR-3651	48.07	64.46	63.20	58.57	42.54	43.17	40.42	42.05	0.72	0.0848
hsa-miR-15a	149.27	141.23	141.23	143.91	119.76	93.99	97.11	103.62	0.72	0.0285
hsa-miR-10b	585.20	463.62	541.38	530.07	398.96	322.66	426.17	382.60	0.72	0.0362
hsa-miR-214	463.62	656.47	762.30	627.46	279.98	541.38	541.38	454.25	0.72	0.2332
hsa-miR-20a	109.46	105.11	109.46	108.01	61.61	87.84	85.92	78.46	0.73	0.0689
hsa-miR-196b	292.49	231.07	239.36	254.31	169.06	190.64	195.87	185.19	0.73	0.0530
hsa-miR-149	35.09	27.88	35.09	32.69	22.55	24.82	24.17	23.85	0.73	0.0576
hsa-miR-455-3p	58.92	69.02	93.99	73.98	44.90	64.46	52.70	54.02	0.73	0.1885
hsa-miR-195	28.20	22.55	33.01	27.92	17.23	20.10	25.22	20.85	0.75	0.1423
hsa-miR-342-3p	81.67	61.61	85.92	76.40	55.27	56.41	60.50	57.40	0.75	0.1211
hsa-miR-454	7.71	8.18	12.40	9.43	7.30	7.44	6.66	7.13	0.76	0.2618
hsa-miR-377	33.01	27.75	27.02	29.26	19.90	22.14	24.58	22.21	0.76	0.0437
hsa-miR-19b	185.55	216.04	181.76	194.45	151.19	144.76	151.19	149.05	0.77	0.0479
hsa-miR-3663-3p	49.82	38.72	34.42	40.99	38.55	27.88	28.95	31.79	0.78	0.1885
hsa-miR-1246	27.75	12.74	10.96	17.15	16.57	14.14	9.47	13.39	0.78	0.5648
hsa-miR-29b-1*	15.98	15.35	16.76	16.03	15.98	11.13	10.96	12.69	0.79	0.1738
hsa-miR-382	22.55	14.36	18.73	18.55	13.21	13.87	17.23	14.77	0.80	0.2515
hsa-miR-493*	46.02	40.42	50.24	45.56	29.26	37.61	42.54	36.47	0.80	0.1381
hsa-miR-193a-3p	14.63	9.21	13.78	12.54	15.19	7.04	8.18	10.14	0.81	0.4814
hsa-miR-199a-5p	902.34	966.96	966.96	945.42	585.20	836.46	902.34	774.67	0.82	0.2153
hsa-miR-1915	92.32	69.91	67.35	76.53	93.99	49.82	44.90	62.90	0.82	0.4938
hsa-miR-3162	59.60	71.84	49.82	60.42	52.70	53.63	43.17	49.84	0.82	0.2367
hsa-miR-17	60.50	60.50	60.50	60.50	41.12	54.65	54.65	50.14	0.83	0.1484
hsa-let-7c	1,603.78	1,603.78	1,770.82	1,659.46	1,380.28	1,380.28	1,380.28	1,380.28	0.83	0.0376
hsa-miR-4271	17.23	10.35	10.01	12.53	14.36	9.80	7.19	10.45	0.83	0.5457
hsa-miR-301a	8.79	6.66	12.74	9.40	7.44	6.33	9.80	7.85	0.84	0.5041
hsa-miR-660	12.74	8.64	10.47	10.62	11.13	8.18	7.44	8.92	0.84	0.3583
hsa-miR-92a	90.53	129.43	149.27	123.08	84.58	112.99	112.99	103.52	0.84	0.3914
hsa-miR-642b	13.87	13.21	10.35	12.48	14.14	9.47	7.97	10.53	0.84	0.4269
hsa-miR-130b	56.41	59.60	61.61	59.21	48.07	46.68	55.27	50.01	0.84	0.0536
hsa-let-7g	836.46	735.53	701.40	757.80	656.47	656.47	656.47	656.47	0.87	0.1297
hsa-miR-425	20.65	17.82	15.98	18.15	13.42	17.82	15.98	15.74	0.87	0.2656

hsa-miR-1260	123.05	87.84	90.53	100.47	87.84	84.58	90.53	87.65	0.87	0.3746
hsa-miR-99a	169.06	176.01	224.22	189.77	131.40	141.23	224.22	165.62	0.87	0.5272
hsa-miR-136	15.35	24.17	36.52	25.35	20.65	18.07	27.75	22.16	0.87	0.6719
hsa-miR-107	336.82	336.82	322.66	332.10	254.45	336.82	279.98	290.42	0.87	0.2260
hsa-miR-25	144.76	169.06	164.25	159.36	123.05	151.19	144.76	139.67	0.88	0.1577
hsa-miR-199a-3p	2,117.75	2,431.51	2,431.51	2,326.92	2,001.53	2,001.53	2,117.75	2,040.27	0.88	0.0976
hsa-let-7f	4,080.50	4,080.50	4,080.50	4,080.50	3,622.04	3,622.04	3,622.04	3,622.04	0.89	
hsa-miR-497	7.97	7.19	8.64	7.93	6.66	7.19	7.30	7.05	0.89	0.1569
hsa-miR-26b	231.07	195.87	176.01	200.98	176.01	195.87	164.25	178.71	0.89	0.3116
hsa-miR-1207-5p	73.67	67.35	51.24	64.09	90.53	44.02	36.52	57.02	0.89	0.7268
hsa-miR-590-5p	16.30	11.85	14.36	14.17	10.96	12.74	14.21	12.64	0.89	0.3954
hsa-miR-30e	31.51	27.02	27.88	28.80	20.31	28.95	28.20	25.82	0.90	0.4066
hsa-miR-151-5p	216.04	254.45	254.45	241.64	195.87	239.36	216.04	217.09	0.90	0.2429
hsa-miR-432	13.42	13.87	16.30	14.53	10.35	14.63	14.36	13.11	0.90	0.4463
hsa-miR-4286	80.05	93.99	58.92	77.65	69.91	82.84	57.87	70.21	0.90	0.5862
hsa-miR-197	19.90	17.23	19.62	18.92	15.67	19.90	16.30	17.29	0.91	0.3678
hsa-let-7b	5,347.17	5,347.17	5,347.17	5,347.17	5,347.17	5,347.17	4,080.50	4,924.95	0.92	0.4226
hsa-miR-337-3p	14.14	12.06	17.23	14.47	10.47	11.52	18.07	13.35	0.92	0.7138
hsa-miR-106b	82.84	85.92	87.84	85.53	73.67	81.67	81.67	79.00	0.92	0.1180
hsa-miR-154	23.41	14.87	13.42	17.23	14.21	15.19	18.73	16.04	0.93	0.7526
hsa-miR-337-5p	30.71	22.14	24.58	25.81	24.17	21.00	27.38	24.18	0.94	0.6347
hsa-miR-27a	1,152.72	1,039.44	1,152.72	1,114.96	902.34	1,039.44	1,214.25	1,052.01	0.94	0.5707
hsa-miR-34b*	29.78	24.82	25.22	26.61	27.88	24.58	23.41	25.29	0.95	0.5612
hsa-miR-28-5p	85.92	112.99	119.76	106.23	97.11	102.15	105.11	101.46	0.96	0.6932
hsa-miR-98	51.24	55.27	73.67	60.06	59.60	52.70	61.61	57.97	0.97	0.7988
hsa-miR-130a	541.38	521.09	585.20	549.23	521.09	485.40	585.20	530.56	0.97	0.6246
hsa-miR-369-5p	10.72	7.30	6.66	8.23	5.92	6.86	11.13	7.97	0.97	0.9070
hsa-miR-34a	762.30	585.20	656.47	667.99	735.53	701.40	521.09	652.67	0.98	0.8649
hsa-miR-101	12.40	4.23	6.33	7.65	7.04	5.44	10.35	7.61	0.99	0.9890
hsa-miR-24-1*	11.52	14.21	19.90	15.21	12.74	13.42	19.28	15.14	1.00	0.9846
hsa-miR-720	966.96	1,152.72	902.34	1,007.34	966.96	1,214.25	836.46	1,005.89	1.00	0.9919
hsa-let-7a	6,475.20	6,475.20	6,475.20	6,475.20	6,475.20	6,475.20	6,475.20	6,475.20	1.00	
hsa-miR-21	27,162.93	27,162.93	27,162.93	27,162.93	27,162.93	27,162.93	27,162.93	27,162.93	1.00	
hsa-miR-125a-5p	195.87	181.76	169.06	182.23	185.55	209.99	158.37	184.64	1.01	0.8952
hsa-miR-1281	4.68	8.79	8.34	7.27	5.76	8.64	7.71	7.37	1.01	0.9520

hsa-miR-299-5p	55.27	53.63	56.92	55.27	52.13	56.92	59.60	56.22	1.02	0.7206
hsa-miR-93	69.02	78.49	75.51	74.34	58.92	85.92	82.84	75.89	1.02	0.8759
hsa-let-7d	504.80	504.80	521.09	510.23	541.38	585.20	463.62	530.07	1.04	0.6345
hsa-miR-26a	521.09	485.40	426.17	477.55	504.80	504.80	485.40	498.33	1.04	0.5342
hsa-miR-361-3p	9.80	8.34	8.18	8.77	10.72	8.48	8.34	9.18	1.05	0.6867
hsa-miR-365	239.36	185.55	231.07	218.66	231.07	216.04	239.36	228.82	1.05	0.6178
hsa-let-7i	701.40	762.30	735.53	733.08	701.40	902.34	735.53	779.76	1.06	0.5354
hsa-miR-487b	33.60	30.71	29.78	31.36	27.38	35.81	37.61	33.60	1.07	0.5612
hsa-miR-939	5.76	9.80	5.44	7.00	13.78	4.68	4.23	7.56	1.08	0.8795
hsa-miR-19a	27.88	28.95	30.71	29.18	28.20	32.16	35.09	31.82	1.09	0.3187
hsa-miR-127-3p	105.11	84.58	102.15	97.28	105.11	90.53	123.05	106.23	1.09	0.4811
hsa-miR-151-3p	38.55	41.60	48.07	42.74	41.60	51.24	48.07	46.97	1.10	0.3489
hsa-miR-381	38.72	35.09	32.16	35.33	30.71	38.55	47.28	38.85	1.10	0.5496
hsa-miR-30e*	13.78	14.14	21.00	16.31	16.76	15.35	21.79	17.97	1.10	0.6165
hsa-miR-27b	1,380.28	1,380.28	1,473.44	1,411.33	1,473.44	1,603.78	1,603.78	1,560.33	1.11	0.0552
hsa-miR-132*	4.23	2.83	4.23	3.77	5.44	4.23	2.83	4.17	1.11	0.6781
hsa-miR-4317	7.44	7.97	7.04	7.48	9.47	8.34	7.04	8.28	1.11	0.3762
hsa-miR-320d	151.19	224.22	190.64	188.68	216.04	224.22	190.64	210.30	1.11	0.4265
hsa-miR-324-5p	47.28	38.55	46.02	43.95	46.68	50.24	51.24	49.39	1.12	0.1743
hsa-miR-1280	14.36	16.30	16.57	15.75	21.79	16.76	14.63	17.73	1.13	0.4549
hsa-miR-362-5p	7.19	4.68	5.92	5.93	8.79	6.66	4.68	6.71	1.13	0.6115
hsa-miR-758	6.33	5.76	7.44	6.51	4.23	5.92	12.06	7.40	1.14	0.7451
hsa-miR-23a	1,770.82	2,117.75	2,330.09	2,072.88	2,431.51	2,330.09	2,330.09	2,363.89	1.14	0.2125
hsa-miR-22	2,001.53	2,001.53	2,001.53	2,001.53	2,330.09	2,117.75	2,431.51	2,293.11	1.15	0.0875
hsa-miR-320e	119.76	164.25	158.37	147.46	181.76	176.01	149.27	169.02	1.15	0.2842
hsa-miR-22*	19.62	19.62	18.40	19.21	21.00	17.23	27.88	22.03	1.15	0.4610
hsa-miR-654-3p	52.13	46.68	53.63	50.82	56.92	48.07	69.91	58.30	1.15	0.3610
hsa-miR-103	398.96	447.53	398.96	415.15	463.62	521.09	447.53	477.41	1.15	0.0934
hsa-miR-30c	67.35	46.02	59.60	57.66	78.49	57.87	63.20	66.52	1.15	0.3697
hsa-miR-132	28.47	18.40	18.07	21.64	23.41	31.51	20.31	25.07	1.16	0.5121
hsa-miR-193b	75.51	75.51	99.33	83.45	85.92	97.11	109.46	97.50	1.17	0.2516
hsa-miR-409-3p	36.52	32.16	33.60	34.09	28.47	41.60	49.82	39.96	1.17	0.4463
hsa-miR-361-5p	71.84	81.67	97.11	83.54	102.15	92.32	99.33	97.93	1.17	0.1799
hsa-miR-152	40.42	28.47	28.47	32.45	35.81	38.72	39.84	38.12	1.17	0.2882
hsa-miR-379	37.61	34.42	41.12	37.72	40.42	40.42	52.13	44.32	1.18	0.2290
hsa-let-7e	735.53	902.34	1,039.44	892.43	1,039.44	966.96	1,152.72	1,053.04	1.18	0.2086

hsa-miR-221*	34.42	23.41	28.20	28.67	38.72	24.17	38.72	33.87	1.18	0.4286
hsa-miR-30b	117.71	73.67	71.84	87.74	117.71	99.33	93.99	103.67	1.18	0.4114
hsa-miR-3653	8.48	8.48	9.80	8.92	7.97	10.35	13.42	10.58	1.19	0.4045
hsa-miR-940	24.17	28.20	31.51	27.96	44.02	28.47	27.02	33.17	1.19	0.4472
hsa-miR-4324	35.81	36.52	42.54	38.29	51.24	39.84	46.02	45.70	1.19	0.1440
hsa-miR-29a	2,330.09	1,770.82	1,380.28	1,827.06	2,117.75	2,431.51	2,001.53	2,183.60	1.20	0.3302
hsa-miR-140-3p	87.84	90.53	209.99	129.45	141.23	114.44	209.99	155.22	1.20	0.6318
hsa-miR-23b	1,214.25	1,214.25	1,603.78	1,344.09	1,603.78	1,473.44	1,770.82	1,616.01	1.20	0.1666
hsa-miR-24	1,473.44	1,473.44	1,214.25	1,387.04	1,770.82	1,770.82	1,473.44	1,671.69	1.21	0.0977
hsa-miR-3196	14.87	20.65	15.67	17.06	32.16	16.57	13.78	20.84	1.22	0.5837
hsa-miR-3125	5.44	10.01	7.71	7.72	7.19	10.72	10.47	9.46	1.23	0.3759
hsa-miR-21*	27.38	57.87	47.28	44.18	47.28	61.61	53.63	54.17	1.23	0.3893
hsa-miR-324-3p	63.20	56.92	54.65	58.25	75.51	71.84	67.35	71.57	1.23	0.0189
hsa-miR-3656	9.47	10.96	11.52	10.65	20.10	10.96	8.48	13.18	1.24	0.5501
hsa-miR-128	21.50	21.79	21.79	21.70	18.07	29.78	33.01	26.95	1.24	0.3665
hsa-miR-125b	3,622.04	3,622.04	3,622.04	3,622.04	4,080.50	4,080.50	5,347.17	4,502.72	1.24	0.1723
hsa-miR-186	12.06	7.04	8.48	9.19	12.06	8.79	13.87	11.57	1.26	0.3215
hsa-miR-1260b	64.46	63.20	69.91	65.85	81.67	80.05	87.84	83.19	1.26	0.0056
hsa-miR-320c	97.11	131.40	129.43	119.31	164.25	158.37	131.40	151.34	1.27	0.1007
hsa-miR-320a	41.60	56.41	55.27	51.09	56.41	73.67	64.46	64.85	1.27	0.1169
hsa-miR-1234	14.21	16.76	19.28	16.75	27.75	18.40	17.82	21.32	1.27	0.2927
hsa-miR-320b	102.15	144.76	117.71	121.54	158.37	169.06	141.23	156.22	1.29	0.0906
hsa-miR-4291	11.13	14.63	14.63	13.46	13.87	18.73	19.62	17.41	1.29	0.1497
hsa-miR-199b-5p	209.99	114.44	112.99	145.81	224.22	164.25	181.76	190.08	1.30	0.3111
hsa-miR-148b	27.02	20.31	22.14	23.16	28.95	27.75	34.42	30.37	1.31	0.0658
hsa-miR-148a	43.17	44.02	69.02	52.07	53.63	69.02	84.58	69.08	1.33	0.2397
hsa-miR-423-5p	18.40	18.88	15.19	17.49	21.50	27.02	21.50	23.34	1.33	0.0654
hsa-miR-99b	84.58	97.11	92.32	91.34	109.46	131.40	129.43	123.43	1.35	0.0267
hsa-miR-1305	13.21	20.10	15.35	16.22	24.82	20.31	20.65	21.93	1.35	0.0917
hsa-miR-374a	69.91	43.17	44.02	52.37	63.20	69.91	80.05	71.05	1.36	0.1559
hsa-miR-574-3p	44.90	35.81	38.55	39.75	49.82	55.27	56.92	54.00	1.36	0.0159
hsa-miR-374b	41.12	29.78	24.17	31.69	36.52	46.02	50.24	44.26	1.40	0.1251
hsa-miR-1228	10.35	15.19	20.10	15.21	27.02	18.88	18.40	21.43	1.41	0.1924
hsa-miR-30a	164.25	117.71	123.05	135.00	209.99	185.55	176.01	190.52	1.41	0.0422
hsa-miR-1238	6.86	10.47	14.14	10.49	17.82	14.21	12.74	14.92	1.42	0.1688
hsa-miR-30a*	22.14	19.90	27.38	23.14	37.61	30.71	31.51	33.28	1.44	0.0311

hsa-miR-191*	7.04	10.72	13.21	10.32	18.40	14.36	11.85	14.87	1.44	0.1573
hsa-miR-2861	18.88	50.24	17.82	28.98	64.46	41.12	19.90	41.83	1.44	0.4857
hsa-miR-3679-5p	8.64	15.98	7.19	10.60	19.62	15.67	10.72	15.33	1.45	0.2757
hsa-miR-185	21.79	18.73	18.88	19.80	25.22	33.60	28.47	29.10	1.47	0.0470
hsa-miR-222	52.70	51.24	52.70	52.22	71.84	67.35	92.32	77.17	1.48	0.0826
hsa-miR-4306	24.82	25.22	24.82	24.95	39.84	35.09	35.81	36.91	1.48	0.0143
hsa-miR-140-5p	131.40	119.76	279.98	177.05	239.36	149.27	398.96	262.53	1.48	0.3987
hsa-miR-30d	61.61	48.07	56.41	55.36	99.33	75.51	71.84	82.23	1.49	0.0713
hsa-miR-224	53.63	39.84	37.61	43.69	60.50	78.49	56.41	65.13	1.49	0.0691
hsa-miR-575	11.85	31.51	23.41	22.25	33.01	33.01	33.60	33.20	1.49	0.1947
hsa-miR-29b	322.66	279.98	185.55	262.73	485.40	398.96	292.49	392.28	1.49	0.1402
hsa-miR-3198	16.57	27.38	21.50	21.82	31.51	34.42	32.16	32.70	1.50	0.0641
hsa-miR-1225-5p	46.68	58.92	57.87	54.49	129.43	58.92	58.92	82.42	1.51	0.3564
hsa-miR-638	18.73	54.65	22.55	31.98	80.05	42.54	22.55	48.38	1.51	0.4710
hsa-miR-29c	254.45	149.27	114.44	172.72	322.66	231.07	231.07	261.60	1.51	0.1694
hsa-miR-409-5p	10.96	7.71	7.97	8.88	11.52	13.78	16.57	13.96	1.57	0.0534
hsa-miR-4299	50.24	99.33	84.58	78.05	149.27	119.76	102.15	123.73	1.59	0.0847
hsa-miR-221	485.40	398.96	447.53	443.96	762.30	447.53	966.96	725.59	1.63	0.2006
hsa-miR-331-3p	93.99	102.15	105.11	100.42	144.76	181.76	169.06	165.20	1.65	0.0196
hsa-miR-210	24.58	52.70	46.68	41.32	43.17	123.05	38.55	68.26	1.65	0.4334
hsa-miR-224*	9.21	5.44	4.68	6.44	10.01	12.40	11.52	11.31	1.76	0.0547
hsa-miR-1275	6.66	13.78	9.21	9.89	18.73	20.65	14.87	18.08	1.83	0.0401
hsa-miR-494	99.33	239.36	144.76	161.15	292.49	279.98	336.82	303.10	1.88	0.0587
hsa-miR-4313	5.92	9.47	12.06	9.15	24.58	13.21	15.19	17.66	1.93	0.1203
hsa-miR-1202	28.95	44.90	40.42	38.09	112.99	44.90	73.67	77.19	2.03	0.1806
hsa-miR-484	8.34	6.86	6.86	7.35	14.63	16.30	14.14	15.02	2.04	0.0010
hsa-miR-100	447.53	292.49	336.82	358.95	1,152.72	463.62	701.40	772.58	2.15	0.1720
hsa-miR-4284	158.37	701.40	463.62	441.13	836.46	1,152.72	1,039.44	1,009.54	2.29	0.0475
hsa-miR-513a-5p	10.01	21.50	10.72	14.08	54.65	25.22	18.88	32.92	2.34	0.2236
hsa-miR-1973	8.18	11.52	7.30	9.00	46.02	19.28	29.26	31.52	3.50	0.0982
hsa-miR-483-3p	2.83	5.92	5.76	4.84	34.42	47.28	69.02	50.24	10.39	0.0448

Supplementary Figure S1

A

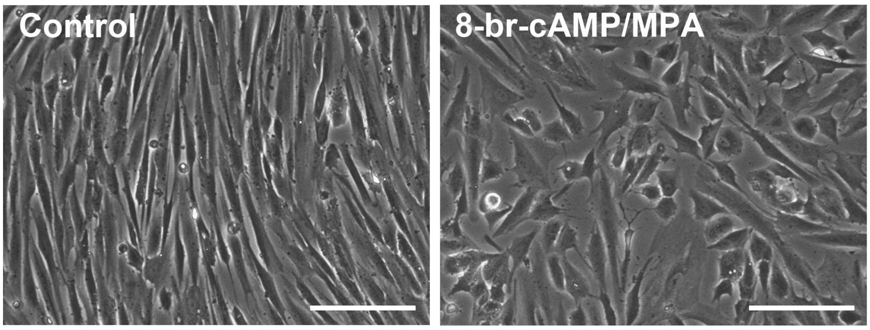

B

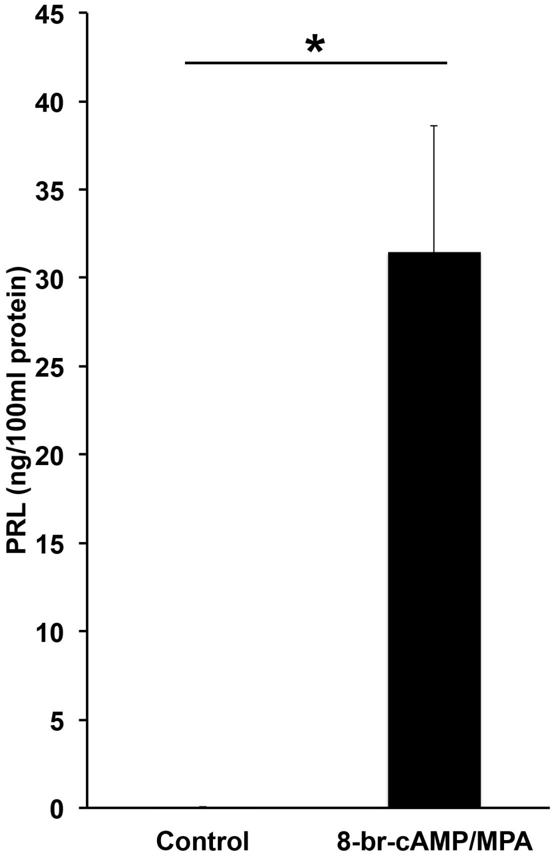

Supplementary Figure S2

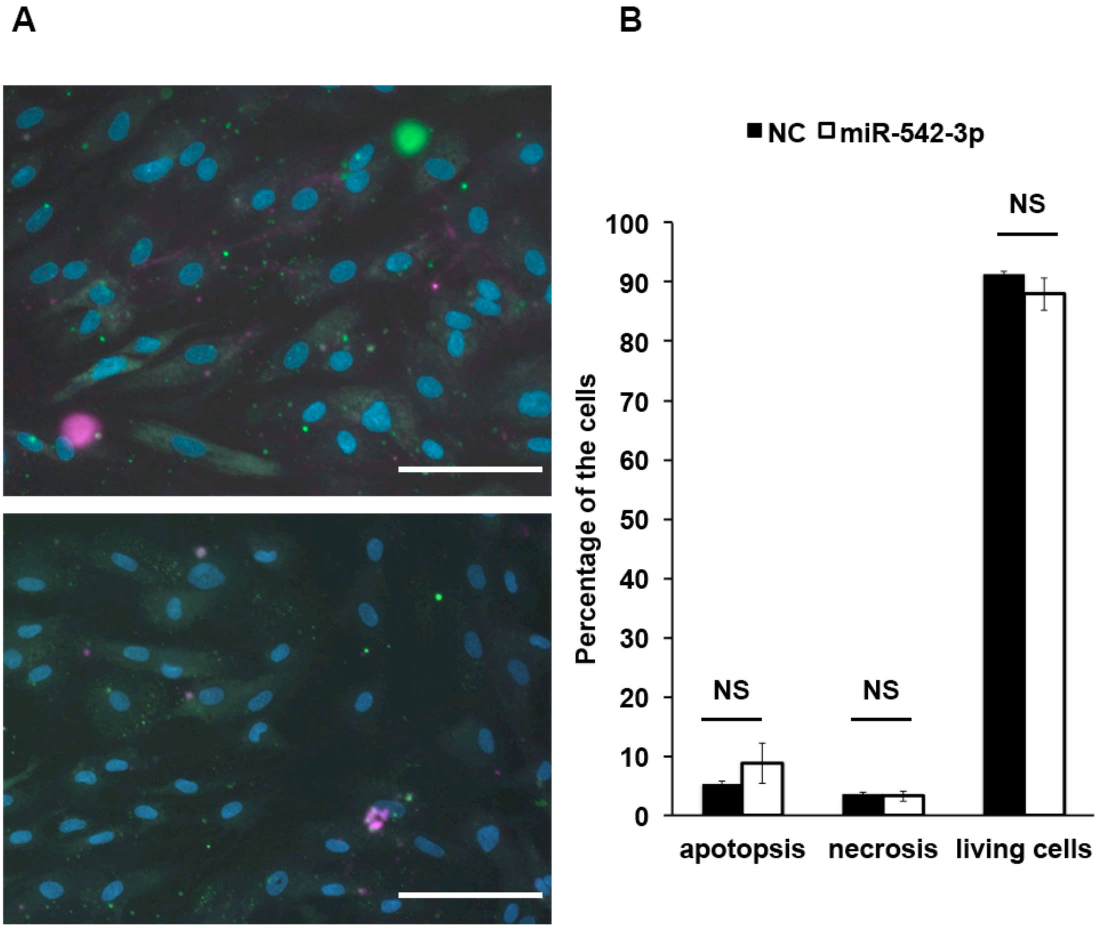

Supplementary Figure S3

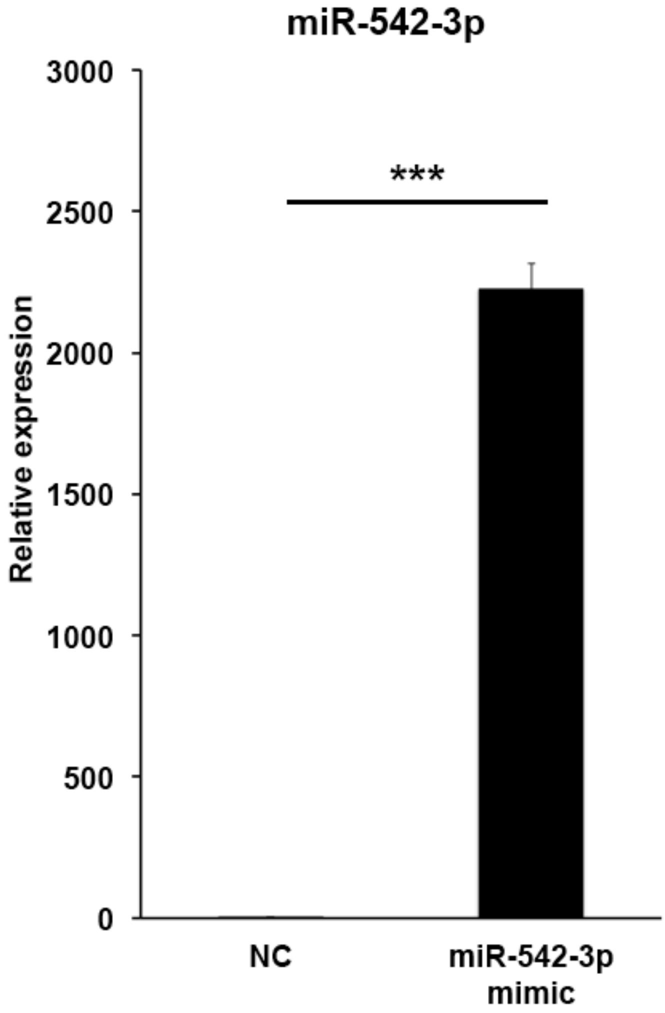

Supplementary Figure S4

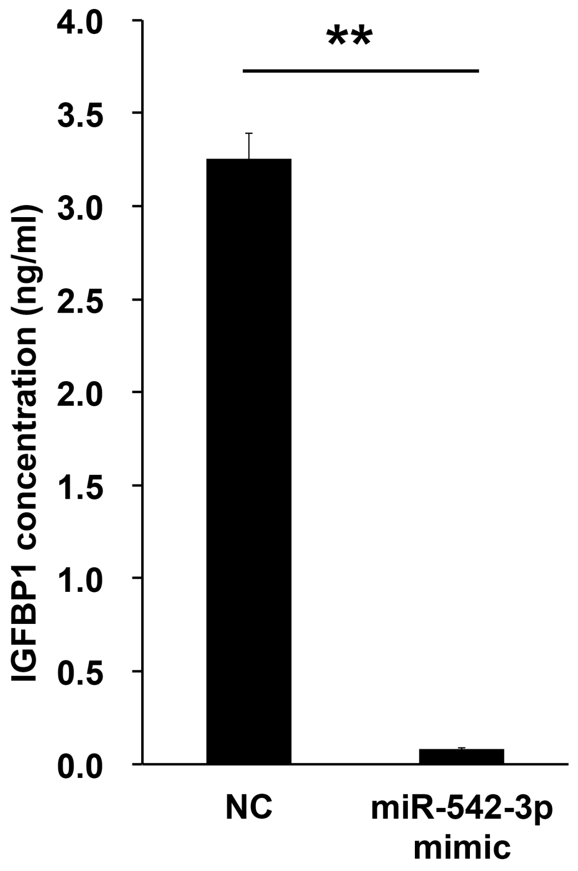

Supplement: Supplementary Information [file srep40001-s1.pdf]
